# Vasa Vasorum Lumen Narrowing in Brain Vascular Hyalinosis in Systemic Hypertension Patients Who Died of Ischemic Stroke

**DOI:** 10.3390/ijms21249611

**Published:** 2020-12-17

**Authors:** Sergiy G. Gychka, Nataliia V. Shults, Sofia I. Nikolaienko, Lucia Marcocci, Nurefsan E. Sariipek, Vladyslava Rybka, Tatiana A. Malysheva, Vyacheslav A. Dibrova, Yuichiro J. Suzuki, Alexander S. Gavrish

**Affiliations:** 1 Department of Pathological Anatomy N2, Bogomolets National Medical University, 01601 Kyiv, Ukraine; snikolaenko.nmu.snt@gmail.com (S.I.N.); dibrova03@ukr.net (V.A.D.); alexander.gavrysh@gmail.com (A.S.G.); 2 Department of Pharmacology and Physiology, Georgetown University Medical Center, Washington, DC 20007, USA; ns1015@georgetown.edu (N.V.S.); n.eminesariipek@gmail.com (N.E.S.); rybkavladyslava@gmail.com (V.R.); 3 Department of Biochemical Sciences “A. Rossi Fanelli”, Sapienza University of Rome, 00185 Rome, Italy; lucia.marcocci@uniroma1.it; 4 Department of Neuropathomorphology, Romodanov Neurosurgery Institute, 04050 Kyiv, Ukraine; morpho.neuro@gmail.com

**Keywords:** brain, hyalinosis, ischemic stroke, oxidative stress, vasa vasorum, vascular

## Abstract

Ischemic stroke is a major cause of death among patients with systemic hypertension. The narrowing of the lumen of the brain vasculature contributes to the increased incidence of stroke. While hyalinosis represents the major pathological lesions contributing to vascular lumen narrowing and stroke, the pathogenic mechanism of brain vascular hyalinosis has not been well characterized. Thus, the present study examined the postmortem brain vasculature of human patients who died of ischemic stroke due to systemic hypertension. Hematoxylin and eosin staining and immunohistochemistry showed the occurrence of brain vascular hyalinosis with infiltrated plasma proteins along with the narrowing of the vasa vasorum and oxidative stress. Transmission electron microscopy revealed endothelial cell bulge protrusion into the vasa vasorum lumen and the occurrence of endocytosis in the vasa vasorum endothelium. The treatment of cultured microvascular endothelial cells with adrenaline also promoted the formation of the bulge as well as endocytic vesicles. The siRNA knockdown of sortin nexin-9 (a mediator of clathrin-mediated endocytosis) inhibited adrenaline-induced endothelial cell bulge formation. Adrenaline promoted protein-protein interactions between sortin nexin-9 and neural Wiskott–Aldrich syndrome protein (a regulator of actin polymerization). Spontaneously hypertensive stroke-prone rats also exhibited lesions indicative of brain vascular hyalinosis, the endothelial cell protrusion into the lumen of the vasa vasorum, and endocytosis in vasa vasorum endothelial cells. We propose that endocytosis-dependent endothelial cell bulge protrusion narrows the vasa vasorum, resulting in ischemic oxidative damage to cerebral vessels, the formation of hyalinosis, the occurrence of ischemic stroke, and death in systemic hypertension patients.

## 1. Introduction

Stroke is a leading cause of long-term disability and death worldwide. In the United States alone, a stroke occurs every 40 s and stroke-induced death occurs every four minutes. Every year, more than 795,000 Americans have a stroke, resulting in 140,000 deaths [1,2,3]. By 2030, an additional 3.4 million U.S. adults are expected to have suffered a stroke (a 20.5% increase in prevalence from 2012) [4,5]. Approximately 87% of all stroke incidences are ischemic strokes, which occur when a blood vessel supplying the brain is obstructed. Systemic hypertension is the most important risk factor for the development of ischemic stroke [1,6]. High blood pressure promotes the alteration of the vascular wall and increases the incidence of ischemic stroke and subsequent neurodegeneration [7,8,9,10].

One important lesion that contributes to the lumen narrowing of the brain vessels is vascular hyalinosis, which refers to the thickening of the vascular wall due to deposits of homogeneous hyaline materials [11,12]. The pathogenesis of the formation of hyaline includes the infiltration of plasma proteins such as apolipoprotein E (ApoE), α2-macroglobulin, fibrinogen, and immunoglobulin G into the vascular wall [13,14]. The accumulation of these plasma components alters the structural components of the vessel wall through the formation of fibrinoid lesions and causes its hyalinization [15,16,17].

Thus, the reversal and ultimately prevention of brain vascular hyalinosis in systemic hypertension patients have the therapeutic potential to reduce the incidence of ischemic stroke. However, the mechanism of brain vascular hyalinosis is not well understood. While the involvement of oxidative stress in various pathological features of stroke has been documented [18], whether oxidative stress occurs in hyalinosis lesions is unknown. The present study performed detailed histological analyses of the hyalinosis lesions in the postmortem brain tissues of human patients who died of ischemic stroke. We found that brain vascular hyalinosis in human systemic hypertension patients who died of ischemic stroke is associated with the narrowing of the vasa vasorum that supplies blood to the cerebral vascular wall and the occurrence of oxidative stress in both vessel walls as well as brain tissues. We also found that the hyalinosis lesions in the brain of patients with stroke exhibited a “bulge” structure that protrudes from endothelial cells into the lumen of the vasa vasorum that may contribute to the occlusion of the vasa vasorum. The present study further addresses the molecular mechanism of this bulge protrusion process.

## 2. Results

### 2.1. Vasa Vasorum Narrowing in Vascular Hyalinosis Lesions in the Brain of Systemic Hypertension Patients Who Died of Ischemic Stroke

**Figure 1:**
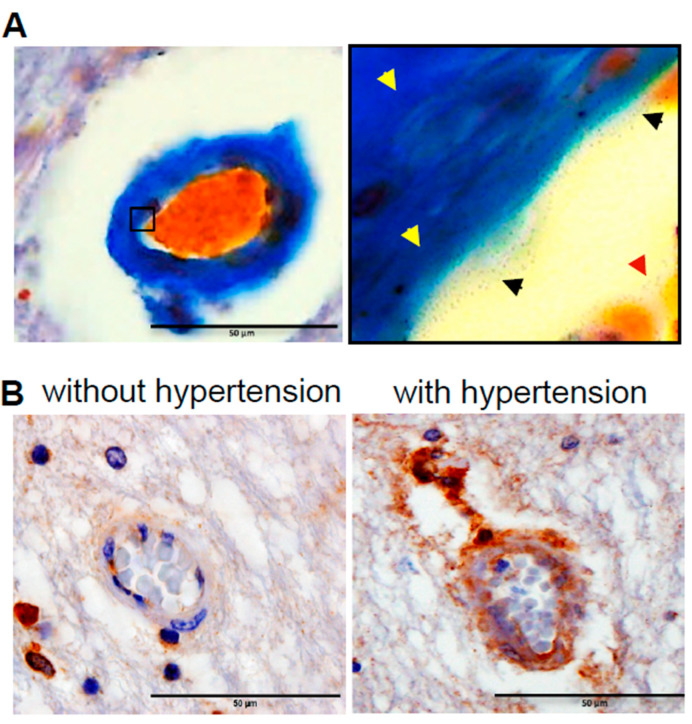
Vascular hyalinosis in systemic hypertension patients who died of ischemic stroke. (**A**) Representative Zerbino–Lukasevich staining of patients with systemic hypertension who died of ischemic stroke. The left panel shows the infiltration of plasma fibrin into the vessel wall. The right panel (magnified, ×1000) shows the fibrin in the blood, at the vessel surface, and in the vessel wall. (**B**) Representative immunohistochemistry results using the ApoE antibody in patients who died of ischemic stroke without hypertension or with hypertension. Positive ApoE stains in the intima, media and adventitia layer were found only in the with hypertension group. Magnifications, ×400. Scale bars, 50 μm. Representative images of *N* = 8 patients per group.

Hyalinosis occurs in the brain vasculature in late-stage systemic hypertension preceding ischemic stroke. Hyalinosis is characterized by the death of vascular cells, ultimately leading to the infiltration and accumulation of plasma proteins, forming glass-like materials that occlude the blood vessel lumen and interfere with the gas exchange between blood and brain tissue. The Zerbino–Lukasevich staining results shown in Figure 1A demonstrate the fibrin infiltration into the vascular wall from the lumen during vascular wall hyalinization. The magnified image in Figure 1A (right panel) shows the presence of fibrins in the blood plasma, the precipitation of fibrins at the luminal surface of blood vessels, and infiltration into the vessel wall. ApoE is another protein that infiltrates hyalinosis lesions [13]. Immunohistochemistry for ApoE revealed positive stains in the vascular wall in association with increased wall thickness due to high blood pressure in systemic hypertension patients who died of ischemic stroke (Figure 1B).

**Figure 2:**
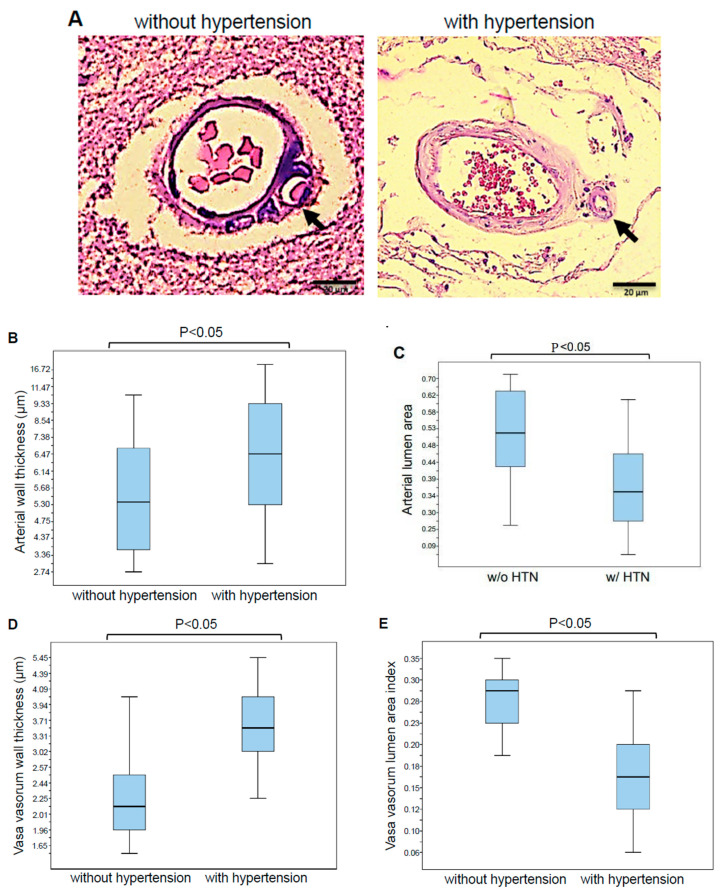
H&E Staining of the vasa vasorum in the brain of patients who died of ischemic stroke. (**A**) Ischemic stroke without hypertension and with hypertension. Representative H&E staining images of *n* = 28 patients per group. Magnifications, ×400. Scale bar, 20 μm. (**B**) Quantification of vessel wall thickness. (**C**) Quantification of the cerebral vascular lumen area index calculated by dividing the internal surface area by the external surface area. *n* = 8 for the without hypertension group and *n* = 6 for the with hypertension group. (**D**) Quantification of vasa vasorum wall thickness. (**E**) Quantification of the vasa vasorum lumen area index calculated by dividing the internal surface area by the external surface area. *n* = 8. Mann–Whitney U test indicated that the values are significantly different at *p* < 0.05.

In these systemic hypertension patients who died of ischemic stroke, hematoxylin and eosin (H&E) staining showed the hyalinosis lesion, with significant cerebral vessel wall thickening (Figure 2A,B) and lumen narrowing (Figure 2A,C). The histological analysis of the adventitia layer of the cerebral vessel revealed that patients who died of ischemic stroke in association with systemic hypertension exhibited significant structural changes of the vasa vasorum. Figure 2A shows representative H&E staining images of the vasa vasorum of cerebral vessels in patients who died of ischemic stroke due to systemic hypertension in comparison with patients without systemic hypertension. In patients with systemic hypertension, the endothelial cells of the intima layer of the vasa vasorum were found to be enlarged and the vasa vasorum media layer was thickened. The morphometric analysis of the vasa vasorum (<25 μm) revealed the wall thickening (Figure 2D) and reduction in the lumen area (Figure 2E) of the vasa vasorum in patients who died of ischemic stroke due to systemic hypertension.

**Figure 3:**
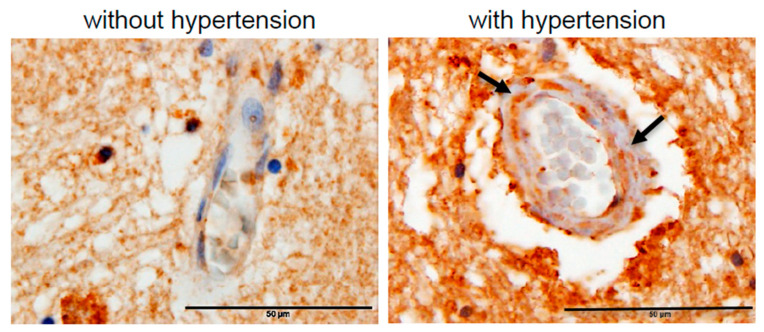
Immunohistochemistry for MDA. Representative immunohistochemistry results using the MDA antibody to assess the levels of oxidative stress in the brain of patients who died of ischemic stroke without hypertension or with hypertension. In patients with hypertension, the intense MDA expression is visible in the cerebral vessel walls (arrows) and the surrounding neuronal tissues. Magnifications ×400. Scale bars, 50 μm. Representative images of *n* = 8 patients per group.

Since the vasa vasorum supplies blood to the walls of cerebral blood vessels (>50 μm), its narrowing may contribute to the initiation and/or progression of cerebral vessel hyalinosis by limiting blood flow as well as oxygen delivery to the brain, thereby promoting oxidative stress. Indeed, immunohistochemistry using the antibody against malondialdehyde (MDA), a lipid peroxidation product, showed that patients who died of ischemic stroke with systemic hypertension, but not those without, had positive MDA staining in both their cerebral vessel walls and their neuronal tissues (Figure 3).

### 2.2. Endothelial Cell Bulge Protrusion into the Vasa Vasorum in the Vascular Hyalinosis Lesions in the Brain of Systemic Hypertension Patients Who Died of Ischemic Stroke

**Figure 4:**
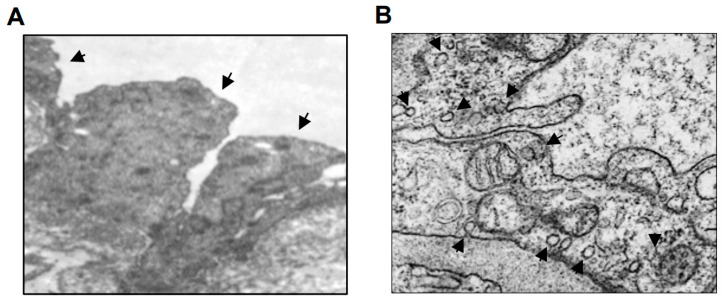
TEM images of the vasa vasorum of the hyalinosis lesions of the brain vessels of systemic hypertension patients. Brain tissues from systemic hypertension patients who underwent neurosurgery to remove the hematoma to treat hemorrhagic stroke were analyzed by TEM. (**A**) Arrows indicate the presence of the endothelial cell bulge. Magnification, ×5600. (**B**) Arrows indicate the presence of endocytic vesicles. Magnification, ×22,000. Representative images of *n* = 6 patients.

Our detailed examinations of the hyalinosis lesions in brain tissues collected from human patients who died of ischemic stroke led us to realize that endothelial cell structural changes contribute to the occlusion of the vasa vasorum due to the formation of a “bulge” structure that protrudes from endothelial cells into the lumen of the vasa vasorum (Figure 2A). We formulated a novel hypothesis that the occlusion of this small vessel network supplying blood to cerebral vascular wall tissues contributes to the ischemic damage to the vascular cells and the formation of hyalinosis. This endothelial cell bulge structure was also visualized by transmission electron microscopy (TEM) in the vasa vasorum in the hyalinosis lesions of the brain vessels of systemic hypertension patients who underwent neurosurgery to remove the hematoma to treat hemorrhagic stroke (Figure 4A). Fresh samples from these patients who underwent surgery provided high quality TEM results that could not be obtained using postmortem tissues. Samples from these hemorrhagic stroke patients with systemic hypertension provided the evidence of the bulge protrusion like in ischemic stroke patients with systemic hypertension. In the vasa vasorum endothelial cells of the brain vasculatures of these patients, we also noted the formation of endocytic vesicles (Figure 4B).

**Figure 5:**
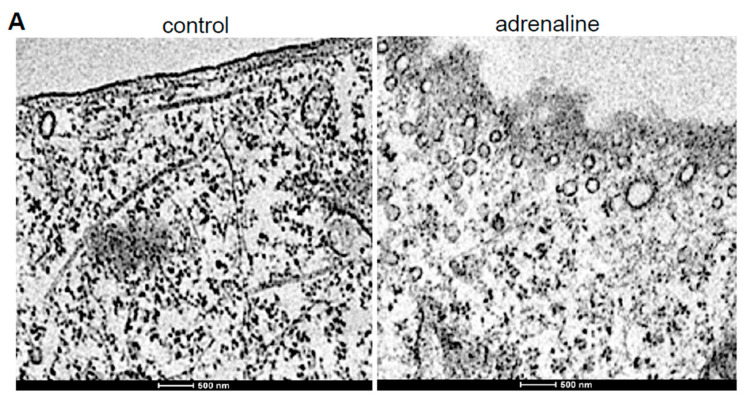

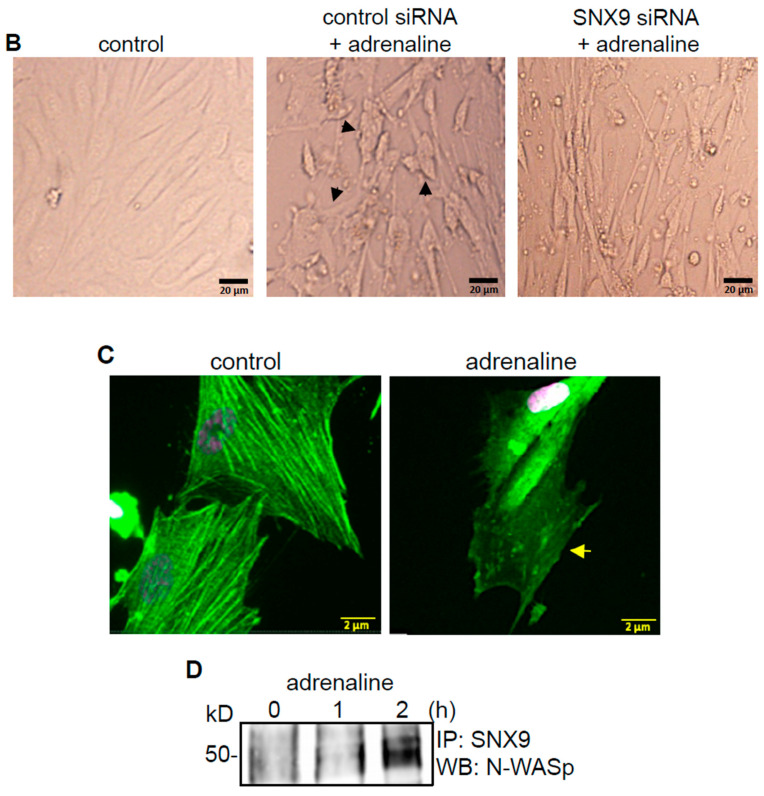
Studies of cultured human microvascular endothelial cells. (**A**) TEM images of the control cells and cells exhibiting the adrenaline-induced formation of endocytic vesicles. Cells were treated with adrenaline at 10 μM for 4 h. Magnifications, ×13,000. (**B**) siRNA knockdown of SNX9 inhibits the adrenaline-induced bulge formation. Cells were treated with siRNA for 2 days and then with adrenaline at 10 μM for 4 h. Cells were observed under light microscopy. Arrows indicate the bulge formation. Magnifications, ×400. (**C**) Immunofluorescence staining of actin in untreated control and cells treated with adrenaline (10 μM; 4 h). The arrow indicates the reorganization of actin filaments. Magnifications, ×1000. (**D**) Adrenaline promotes SNX9–N-WASp interactions. Cells were treated with 10 μM adrenaline for 0, 1 or 2 h. Cell lysates were subjected to immunoprecipitation (IP) with mouse SNX9 IgG followed by Western blotting (WB) with rabbit N-WASp IgG.

Further, in cultured human microvascular endothelial cells, the treatment with adrenaline, an important contributor of vascular changes associated with systemic hypertension [19,20], promoted the formation of endocytic vesicles, as visualized by TEM (Figure 5A), as well as the bulge-like structure, as observed by light microscopy (Figure 5B). Immunofluorescence staining showed that the bulge formation induced by adrenaline was associated with the reorganization of actin filaments (Figure 5C). We hypothesized that adrenaline activated clathrin-mediated endocytosis that, in turn, elicited protein-protein interactions between sortin nexin 9 (SNX9; a mediator of endocytosis) and neural Wiskott–Aldrich syndrome protein (N-WASp; a regulator of actin polymerization) [21,22,23], resulting in structural changes to the actin filament in endothelial cells. In support of this, we found that the siRNA knockdown of SNX9 inhibited adrenaline-induced bulge formation (Figure 5B) and that adrenaline promoted SNX9–N-WASp interactions (Figure 5D).

**Figure 6:**
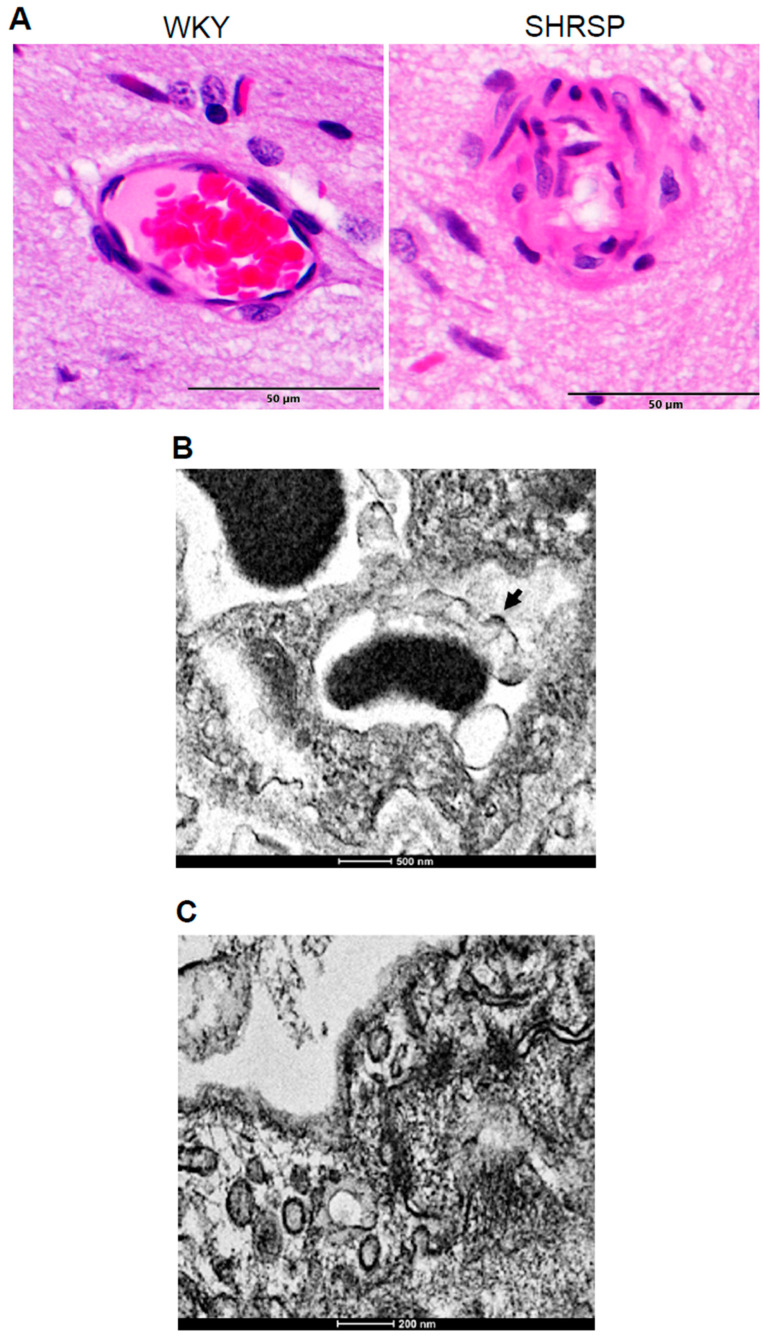
Brain vascular hyalinosis in SHRSP rats. (**A**) H&E staining images of the brain vessels of Wistar–Kyoto (WKY) control rats and SHRSP rats at 8 weeks of age. Magnifications, ×400. (**B**) TEM image of the endothelial cell bulge protrusion into the lumen of the vasa vasorum in SHRSP rats. Magnification, ×14,500. (**C**) TEM image of endocytic vesicles in the endothelium of the vasa vasorum of SHRSP rats. Magnification, ×18,000. Representative images of *n* = 4 rats per group.

Spontaneously hypertensive stroke-prone rats (SHRSP) that have been studied for more than 40 years showed various pathological lesions similar to human patients who suffer from systemic hypertension and stroke [24]. We found that these rats also exhibited lesions indicative of brain vascular hyalinosis at 8 weeks of age (Figure 6A). In the TEM images of the brain vessels of these rats, we also observed the endothelial cell protrusion into the lumen of the vasa vasorum (Figure 6B) as well as endocytosis in vasa vasorum endothelial cells (Figure 6C).

## 3. Discussion

In the postmortem brain tissues obtained from patients who died of ischemic stroke due to systemic hypertension, we performed histological analyses to define the occurrence of brain vascular hyalinosis. In such lesions, plasma proteins were deposited in the vessel wall and the indication of oxidative stress was detected. We also found that the lumen of the vasa vasorum that supplies blood to the wall of the cerebral vessels narrowed.

According to our result, lipid peroxidation products reside in the hyalinosis lesions in both vessels and brain tissues. Immunohistochemistry using the MDA antibody could stain free MDA or MDA–protein adducts. Thus, it is not yet clear whether lipid peroxidation occurs in the membrane systems associated with hyalinosis lesions or whether MDA-bound protein migrates to these lesions. Plasma proteins that are infiltrated may be oxidized and thus form oxidation-dependent aggregates, which may then be accumulated as hyaline.

The major finding of this study is that in addition to cerebral arterial vessels, the vasa vasorum is narrowed. These results suggest that ischemia-reperfusion injury due to the narrowing of the vasa vasorum triggers oxidative stress damage to cerebral vascular walls, resulting in the subsequent occlusion of cerebral arteries and ischemic insult to brain tissues.

**Figure 7:**
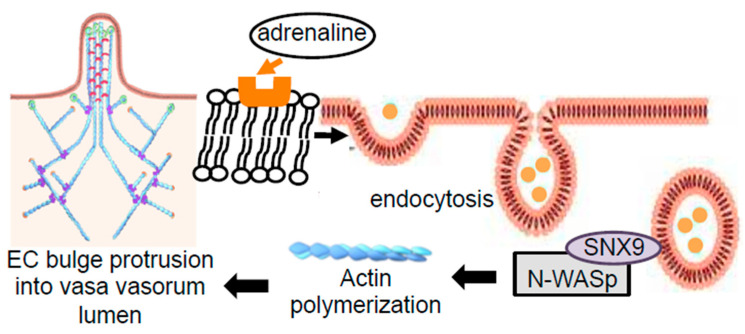
Scheme depicting the proposed mechanism of the endothelial cell (EC) bulge protrusion into the vasa vasorum lumen. In systemic hypertension patients, adrenaline promotes the endocytosis-dependent SNX9–N-WASp interaction that activates the actin polymerization and the EC bulge protrusion into the lumen of the vasa vasorum, resulting in narrowing the lumen of the vasa vasorum.

The present study provides evidence to support the mechanistic hypothesis depicted in Figure 7 that the endothelial cell cytoskeletal rearrangement forms a bulge-like structure in the lumen of the vasa vasorum through adrenaline-induced endocytosis and SNX9–N-WASp interaction-mediated actin reassembly, contributing to the occlusion of these small vessels that supply oxygen to the cerebral vascular wall.

In summary, this study provided histological characterizations of human brain vascular hyalinosis in systemic hypertension patients who died of ischemic stroke. We propose that vasa vasorum narrowing results in oxidative stress to cerebral vessels, contributing to the formation of brain vascular hyalinosis, the occurrence of ischemic stroke, further oxidative stress to neuronal cells, and eventual death in systemic hypertension patients. Further understanding this pathological lesion is critical to developing new and effective therapeutic strategies to reduce the risk of ischemic stroke in systemic hypertension patients, which comprise a large population worldwide.

## 4. Materials and Methods

### 4.1. Autopsy Brain Tissues from Patients

Postmortem brain tissues were collected from 28 patients with a history of systemic hypertension and who died of ischemic stroke (mean age 70; age range 53–82; 64% female) and 28 patients who died of ischemic stroke without systemic hypertension (mean age 80; age range 70–89; 57% female). The tissues were taken from the perifocal zone of the ischemic infarct in the region of the middle cerebral artery of the frontal lobe. Clinical studies were approved by the regional committee for medical research ethics in Kiev, Ukraine (ethical code: 81, 11 March 2016) and performed under the Helsinki Declaration of 1975 revised in 2013 or comparable ethical standards. The participants provided written informed consent.

### 4.2. Experimental Animals

Male and female SHRSP rats (originally isolated from Wistar–Kyoto rats [25]) and Wistar–Kyoto rats as controls were purchased from Charles River Laboratories International, Inc. (Wilmington, MA, USA). The animals were fed normal rat chow. The Georgetown University Animal Care and Use Committee approved all the animal experiments (Protocol # 2019-0045 approved on 27 August 2019), and the investigation conformed to the National Institutes of Health Guide for the Care and Use of Laboratory Animals.

### 4.3. Histological Measurements

The sections were resected to be approximately 10 μm in thickness and immersed in 10% buffered formalin at room temperature. The fixed tissue samples were paraffin embedded, sectioned at 6 μm with a microtome, mounted on glass slides, and analyzed histochemically and immunohistochemically. The tissue sections were subjected to H&E staining to determine the general morphology of the brain vessels, Zerbino–Lukasevich staining to detect the presence of fibrin to assess the vascular lesions, and immunohistochemistry using anti-ApoE and anti-MDA antibodies (Abcam, Cambridge, UK). Vessels smaller than 25 μm or larger than 150 μm in external diameter were excluded from the analysis.

### 4.4. TEM Analysis

Brain tissues were collected from patients who underwent neurosurgery and immediately fixed in a solution containing 4% paraformaldehyde and 0.5% glutaraldehyde/0.2 M cacodylate. The samples were then post-fixed with 1% osmium tetroxide and embedded in EmBed812. Ultrathin sections were stained with uranyl acetate and lead citrate and examined using a Philips EM-400T transmission electron microscope at 80 kV with the TIA software.

### 4.5. Cell Culture

Human microvascular endothelial cells were purchased from ScienCell Research Laboratories (Carlsbad, CA, USA) and cultured in accordance with the manufacturer’s instructions in 5% CO_2_ at 37 °C. Cells in passages 3–6 were used. The siRNA experiments were performed using the Santa Cruz Biotechnology (Dallas, TX, USA) system in accordance with the manufacturer’s instructions. Briefly, cells grown on six-well plates were incubated with 1 µg SNX9 siRNA (Catalog # sc-61597) or Control siRNA (Catalog # sc-37007) and siRNA transfection reagent in siRNA transfection medium for 5 h. An equal volume of growth medium containing twice the normal serum, growth supplements, and antibiotics was added, and the cells were grown for 2 days.

### 4.6. Immunoprecipitation and Western Blotting

Cell lysates were immunoprecipitated with the mouse polyclonal anti-SNX9 antibody (Santa Cruz) and SureBeads Protein G Magnetic Beads (Bio-Rad Laboratories, Hercules, CA, USA) for 1 h at room temperature. Immunoprecipitation using SureBeads was performed in accordance with the manufacturer’s instructions. The samples were electrophoresed through a reducing SDS polyacrylamide gel and electroblotted onto a nitrocellulose membrane. The membranes were blocked and incubated with the antibody to detect N-WASp (MilliporeSigma, Burlington, MA, USA) using horseradish peroxidase-linked secondary antibodies and an Enhanced Chemiluminescence System (GE Healthcare Bio-Sciences, Pittsburgh, PA, USA). Autoradiography was performed using UltraCruz Autoradiography Films (Santa Cruz Biotechnology). The developed films were scanned and the optical densities of the protein bands were quantified using NIH ImageJ.

### 4.7. Statistical Analysis

Statistical analysis was performed with the IBM SPSS Statistics version 23.0 software. Significant differences between two groups were determined by the independent samples Mann–Whitney U test.

## Abbreviations

ApoE	Apolipoprotein E
H&E	Hematoxylin and eosin
MDA	Malondialdehyde
NIH	National Institutes of Health
N-WASp	Neural Wiskott–Aldrich syndrome protein
SHRSP	Spontaneously hypertensive stroke prone
SNX9	Sortin nexin 9
TEM	Transmission electron microscopy
